# Genome-Wide Identification and Expression Analysis of the ALKB Homolog Gene Family in Potato (*Solanum tuberosum* L.)

**DOI:** 10.3390/ijms252010984

**Published:** 2024-10-12

**Authors:** Yan Li, Xuanming Dong, Jianyu Ma, Chenxin Sui, Hongju Jian, Dianqiu Lv

**Affiliations:** 1 Integrative Science Center of Germplasm Creation in Western China (CHONGQING) Science City, Biological Science Research Center, Southwest University, Chongqing 400716, China; 2 College of Agronomy and Biotechnology, Southwest University, Chongqing 400715, China; 3 Key Laboratory of Forest Plant Ecology, Ministry of Education, Northeast Forestry University, Harbin 150040, China

**Keywords:** AlkB homologs, potato, m^6^A RNA methylation, demethylase, abiotic stress

## Abstract

*N*^6^-methyladenosine (m^6^A) is an abundant and pervasive post-transcriptional modification in eukaryotic mRNAs. AlkB homolog (ALKBH) proteins play crucial roles in RNA metabolism and translation, participating in m^6^A methylation modification to regulate plant development. However, no comprehensive investigations have been conducted on *ALKBH* in potato. Here, 11 *StALKBH* family genes were identified in potato and renamed according to BLASTP and phylogenetic analyses following the *Arabidopsis* genome. The characteristics, sequence structures, motif compositions, phylogenetics, chromosomal locations, synteny, and promoter *cis*-acting element predictions were analyzed, revealing distinct evolutionary relationships between potato and other species (tomato and *Arabidopsis*). Homologous proteins were classified into seven groups depending on similar conserved domains, which implies that they possess a potentially comparable function. Moreover, the *StALKBH*s were ubiquitous, and their expression was examined in the various tissues of a whole potato, in which the *StALKBH* genes, except for *StALKBH2,* were most highly expressed in the stolon and flower. Multiple hormone and stress-response elements were found to be located in the promoters of the *StALKBH* genes. Further qRT-PCR results suggest that they may be significantly upregulated in response to phytohormones and abiotic stress (except for cold), and the expression of most of the *StALKBH* genes exhibited positively modulated trends. Overall, this study is the first to report a genome-wide assessment of the *ALKBH* family in potato, providing valuable insights into candidate gene selection and facilitating in-depth functional analyses of ALKBH-mediated m^6^A methylation mechanisms in potato.

## 1. Introduction

AlkB homolog (ALKBH) proteins belong to the dioxygenase superfamily, an important family of specific demethylases that are becoming better known in m^6^A RNA modifications. Over 160 diverse RNA modifications have been identified to date, with *N*^6^-methyladenosine (m^6^A), *N*^1^-methyladenosine (m^1^A), and 5-methylcytidine (m^5^C) being common internal modifications observed in coding RNAs (mRNAs). Among these, m^6^A post-transcriptional modifications most prevalently exist in eukaryotic mRNAs [1,2]. m^6^A methylation modifications mainly involve three types of enzymes in plants: methyltransferases (writers), which include MTA (mRNA adenosine methylase), MTB, FIP37 (FKBP12 INTERACTING PROTEIN 37KD), VIR (VIRILIZER), HAKAI, and FIONA1 (FIO1) [3,4]; demethylases (erasers), such as the *ALKBH* gene family (alkylated DNA repair protein ALKB homologs), which are responsible for removing methyl groups; and recognition enzymes (readers), which bind specifically to m^6^A sites in order to determine the fate of RNA, with most being members of the group of YTH domain-containing proteins [5]. Thus, m^6^A-mediated methylation is a reversibly dynamic equilibrium mechanism that plays an essential role in RNA metabolism, including mRNA stability, splicing, translation efficiency, and nuclear export, by recruiting reader proteins to regulate various biological functions such as growth, development, and the stress response [6,7]. In recent years, m^6^A has been a meaningful and bourgeoning focus of post-transcriptional epigenetic research. For instance, recent reports have demonstrated that m^6^A methylation affects miRNA biogenesis by regulating the secondary structure of miRNA precursors and the recruitment of microprocessor components in *Arabidopsis* [8,9]. However, most studies on m6A are focused on mammals and other crops, and studies on the regulated mechanisms of m^6^A in potatoes are decidedly limited.

The discovery of m^6^A demethylases suggested that m^6^A was a reversible modification, thus accelerating functional research on m^6^A. In 1983, the *ALKBH* genes were first found to eliminate methylation damage in *E. coli* [10]. The demethylases ALKBH5 [11] and FTO [12], both members of the Fe (II)/α-kg-dependent dioxygenase superfamily, were subsequently found in animals. Moreover, it was found that the ALKBH-mediated methylation mechanism can regulate plant growth, development, and stress response via epigenetic modification [5,7]. In *Arabidopsis*, *alkbh10b* mutants were observed to delay the floral transition process, which was accomplished by *ALKBH10B*-mediated m^6^A methylation regulating the key flowering genes FLOWERING LOCUS T (FT), SQUAMOSA PROMOTER BINDING PROTEIN LIKE 3 (SPL3), and SPL9 [13]. It was found that *SlALKBH2*, an mRNA m^6^A demethylase, can bind to the transcript of *SlDML2* and control tomato fruit ripening [14]. Additionally, growing evidence suggests that *ALKBH* gene family members have critical biological functions in abiotic stress responses in plants, especially enhancing plant resistance under abiotic stress conditions such as drought, salt, and extreme temperature. In *Arabidopsis*, it was found that salt stress can promote *AtALKBH9* gene expression in the roots and decrease the expression of *AtALKBH10A* under high-temperature conditions [15]. *alkbh10b* mutants were observed to have a reduced osmotic and salt stress tolerance during seed germination, and they exhibited a salt-hypersensitive phenotype in the seedling stage [16]. A recent study found that *alkbh6* mutants had higher levels of m^6^A methylation and exhibited higher salt, drought, and heat stress tolerance than Col-0, which may decrease ABA signaling-related gene expression via *AtALKBH6*-triggered m^6^A demethylation [17]. In cotton, it was found that the demethylase *GhALKBH10B* can enhance drought tolerance at the seedling stage by reducing the level of m^6^A modification and contributing to the reduction in the mRNA stability of ABA signaling pathway genes and Ca^2+^ signaling-related genes [18]. Moreover, the salt tolerance of *GhALKBH10*-overexpressing *Arabidopsis* decreased, and that of *GhALKBH10*-silenced cotton improved [19]. In summary, considering the expansion of family members and potential functional diversity, it is necessary to conduct a more detailed evolutionary analysis to distinguish whether potential m^6^A-mediated demethylation elements exist.

Potato (*Solanum tuberosum* L.) is an economically important vegetable worldwide, and it possesses the ability to accumulate a large quantity of carbohydrates, making it an essential part of human nutrition. However, no studies have comprehensively or systematically identified the *ALKBH* gene family in potatoes, and their functions have not yet been adequately analyzed. Furthermore, there are few studies on m^6^A methylation modification in potatoes. Recently, it was found that the overexpression of the human m^6^A demethylase *FTO* in potatoes can induce a substantial increase in field yield and biomass of about 50% [20], suggesting that m^6^A methylation has the potential to significantly improve the growth, development, yield production, and cultivation of potato. In the present research, we performed genome-wide identification and structural, evolutionary, expression pattern, and abiotic stress analyses of the *ALKBH* family in the potato genome. Our study is the first to reveal the *ALKBH* family genes of m^6^A demethylase members in potatoes, providing insights for researching their biological functions in the future.

## 2. Results

### 2.1. Genome-Wide Identification of ALKBH Gene Family Members in Potato

To identify the *ALKBH* members in potato, we used 13 *Arabidopsis* and 8 tomato ALKBH protein sequences as queries and screened them against Pfam databases simultaneously. In brief, the results revealed 11 StALKBHs. The results displayed in detail the characteristics of the 11 StALKBHs originating from potato genomic sequences, including gene length, the number of amino acid sequences, molecular weight, isoelectric point (pI), GRAVY, and predicted subcellular localization (Table 1), with nomenclature according to the *Arabidopsis* genome. The deduced protein sequence lengths of the 11 StALKBHs ranged from 253 to 657 aa, and the molecular weights ranged from 29.06 to 72.67 kDa. Among them, seven proteins with <6.5 pI values were acidic, three with >7.5 pI values were alkaline, and one with a pI value between 6.5 and 7.5 was neutral. GRAVY values ranged from −0.17 to −0.64, suggesting that all proteins were hydrophilic. The subcellular localization prediction results revealed that all 11 StALKBH proteins were located in the nucleus.

### 2.2. Chromosomal Location and Collinearity Analysis of ALKBH Family

**Figure 1:**
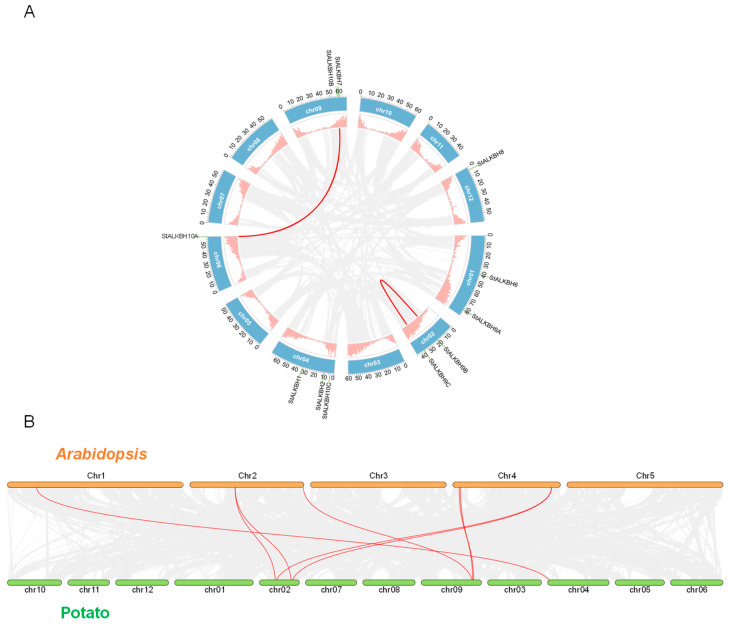
Chromosomal location and collinearity analysis of the *StALKBH* genes in potato. (**A**) Locations of the *StALKBH* genes on potato chromosomes and synteny analysis of the *StALKBH* genes in potato. The gray lines represent the collinearity result of the tomato genome, and the red lines represent the segmental duplication events. (**B**) Synteny analysis of the m^6^A genes between potato and *Arabidopsis*. The gray lines represent the collinearity result between potato and *Arabidopsis* genomes, and the red lines represent the homologous genes.

Regarding the chromosomal location of the *StALKBH* gene family members in the potato genome, they were found to be distributed on six chromosomes, and most were on both ends of the chromosomes. Chromosome 4 (Chr04) contained most of the *StAlKBH* members. Among these, *StAlKBH10B* and *StAlKBH7* were adjacent on Chr09, which may have been caused by a tandem duplication event. The amino acid sequences that resulted from these tandem duplications were highly conserved between the members. Regarding the gene density of the whole potato genome, a large number of genes were located on either ends of the chromosomes; these findings are consistent with the above results. Moreover, besides tandem duplication, segmental duplication was another driving force for gene family evolution. A genome-wide synteny analysis of potatoes revealed two gene pairs—*StAlKBH9B-StAlKBH9C* and *SlALKBH10A-SlALKBH10B*—that were confirmed to result from segmental duplication (Figure 1A). Therefore, both tandem and segmental duplication contributed to the expansion of the *StALKBH* gene family. Additionally, we used Tbtools software to construct the whole-genome synteny relationship between potato and *Arabidopsis*, investigating the WGD (whole-genome duplication) events in the *StALKBH* gene family (Figure 1B). A collinearity analysis showed eight pairs comprising eight potato genes and six *Arabidopsis* genes, and most homo-genes had a one-to-one correspondence between the two species, which suggests that these orthologous pairs existed before the divergence of *Arabidopsis* and potato.

### 2.3. Evolutionary and Sequence Analyses of StALKBH Family

**Figure 2:**
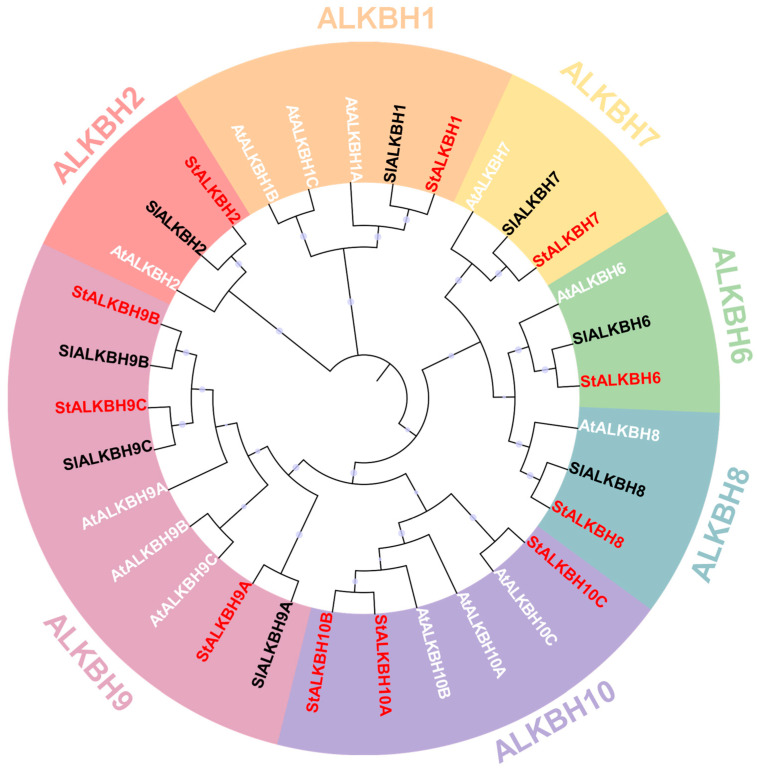
Evolutionary analyses of the *ALKBH* family in potato. A phylogenetic tree of the *ALKBH* family proteins in tomato (Sl, black letters), *Arabidopsis* (At, white letters), and potato (St, red letters), constructed using MEGA10 with the NJ method.

The relationships between other species were examined to demonstrate their evolution. A phylogenetic tree was constructed among *Arabidopsis*, tomato (*Solanum lycopersicum* L.), and potato, as shown in Figure 2. The ALKBH proteins clustered into seven groups (ALKBH1, ALKBH2, ALKBH6, ALKBH7, ALKBH8, ALKBH9, and ALKBH10) following the nomenclature of the *Arabidopsis* genome. Notably, the number of ALKBH proteins varied among the species, with the largest number being found in *Arabidopsis* and the smallest number being found in tomato, which did not contain proteins of the ALKBH10 group. Furthermore, the ALKBH10 group was the largest, comprising nine members that displayed a number of differences between the species, and ALKBH2, ALKBH6, and ALKBH7 consisted of the smallest number of members. In plants, the ALKBH9 and ALKBH10 groups are relatively closely related to the human m^6^A RNA demethylation modification enzyme ALKBH5 [21], and the ALKBH gene family is functionally highly conserved [21,22]. Further validating this perspective, multiple sequence alignment revealed that most members at relative domains were conserved, such as 2OG-FeII_Oxy_2/superfamily and RNA binding, while few members exhibited differences (Figure S1). Regarding the interaction network of the m^6^A-related proteins in potato, the results showed that StALKBHs may combine with other “writer” and “reader” proteins to regulate m^6^A methylation modification (Figure S2). Hence, we next focused on ALKBH9A/B/C and ALKBH10A/B/C in *Arabidopsis* and potato, as ALKBH9 and ALKBH10 subfamily proteins were found to be orthologs of the m^6^A methylase HsALKBH5, indicating that they potentially have more important functions in these candidates. Relative conserved functional sites were found, including protein residues involved in AdoMet interactions and RNA binding, suggesting that *Arabidopsis* and potato may have a similar core heterodimer catalyzing mechanism for removing methylation.

### 2.4. Gene Structure, Conserved Motifs, and Promoter Analysis of StALKBHs

**Figure 3:**
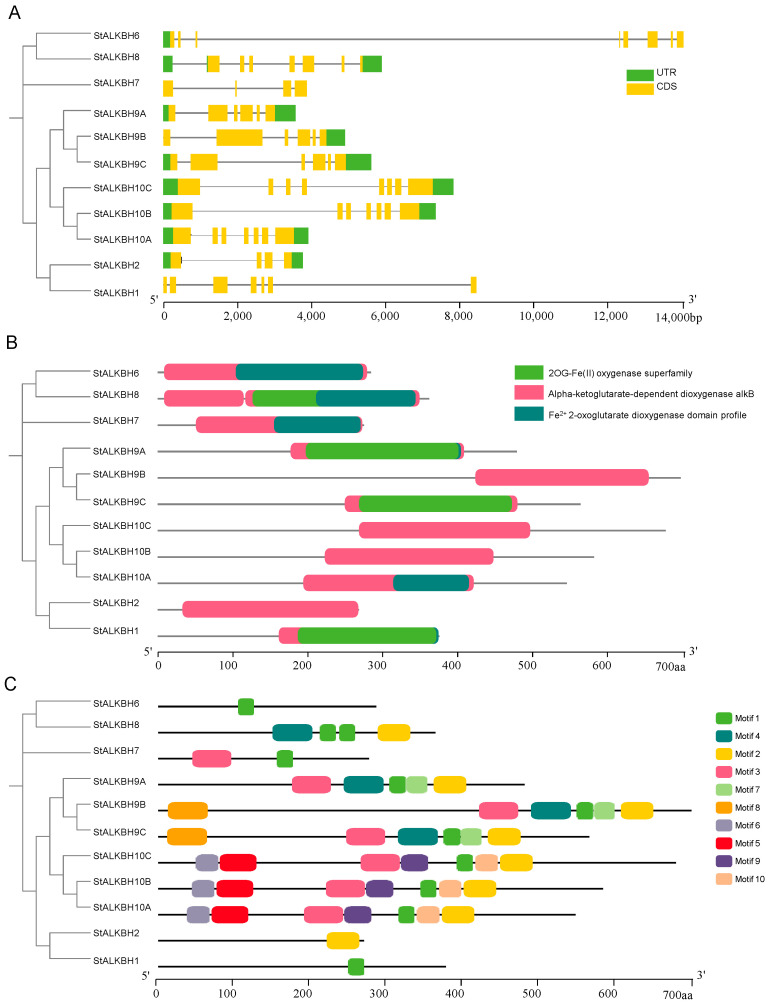
Sequence structure analysis of the *StALKBH* members. (**A**) Gene structures and phylogenetic relationships of *StALKBH*s. The green boxes, black lines, and yellow boxes in the gene structure diagram represent untranslated regions (UTRs), introns, and coding sequences (CDSs), respectively. (**B**) Conserved domain location in the StALKBH proteins, obtained from the PFAM database. (**C**) Comparison of conserved motifs and domain composition of the StALKBHs. Different color boxes indicate different motifs.

During evolution, discrepancies tend to occur in exon/intron structures in coding regions, which determine gene duplication; these result in amino acid-altering substitutions and/or alterations, which subsequently convert the function of genes in order to adapt to different growth conditions [23]. The gene exon/intron structure and conserved motif patterns of *StALKBH*s are exhibited in an NJ tree, which was constructed to examine phylogenetic relationships in order to comprehensively determine family features. According to the results of the phylogenetic analysis, the *StALKBH* family could be grouped into three major clades depending on the similarity of sequences, suggesting a closer evolutionary relationship and a more similar gene structure. For instance, *StALKBH9A/B/C* in the same group contained six exons. Notably, neither *StALKBH1* nor *StALKBH7* contained non-coding regions. These results indicate that the *StALKBH* genes were evolutionarily conserved; additionally, the exon and intron lengths varied, which indicates that the proteins have diverse gene structures. The results of a conserved structural domain analysis revealed that the StALKBH protein sequences contained 2OG-FeII_Oxy_2-related motifs, illustrating that the *StALKBH* candidates belong to the ALKBH superfamily. In addition, the proteins in the same group exhibited similar motif distribution models (Figure 3), among which motif 1 was confirmed to be present in all *StALKBH* genes, except for in *StALKBH2*. Excluding the common motifs, specific motifs were present in specific groups: motifs 5 and 6 were only present in the *StALKBH10A/B/C* group, and motif 7 was only present in the *StALKBH9A/B/C* group. Thus, the *StALKBH* genes in the same subgroup possessed similar compositions in terms of conserved motifs and gene structures, suggesting that StALKBH members in the same cluster may have similar functions.

**Figure 4:**
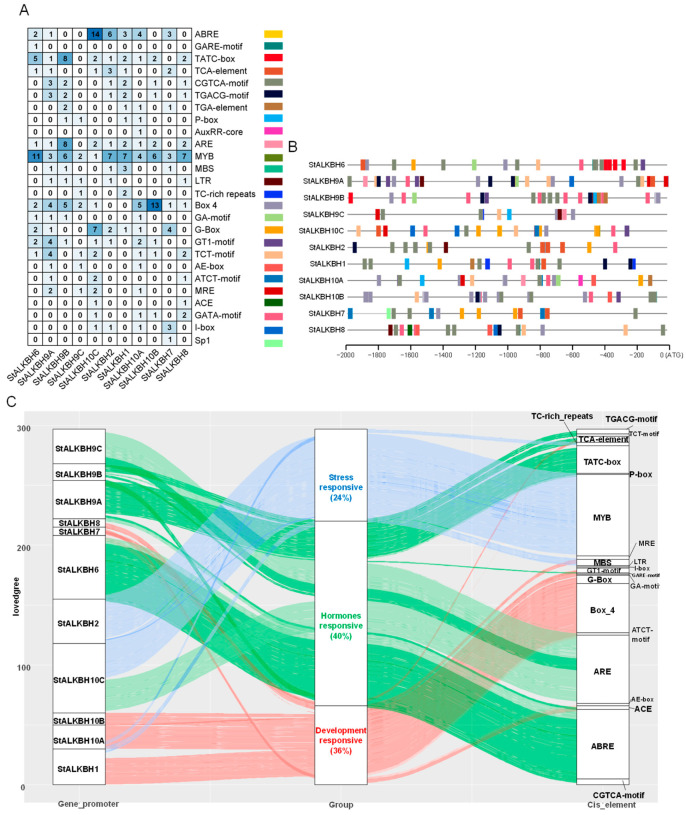
Promoter *cis*-element sequence analysis of the *StALKBH* members. (**A**) Number of *cis*-elements in the *StALKBH* promoters. (**B**,**C**) Various *cis*-elements distributed in the different *StALKBH* promoters; they may have potential functional division. The sequences were obtained from 2000 bp upstream of ATG. Different *cis*-elements are represented with different colors and detailed functional annotations.

To investigate the potential important functions of *StALKBH*s in development and growth, we observed and analyzed the promoters of the *cis*-elements of the 2kb promoter sequence upstream of the start codon (Figure 4). The allocation patterns and numbers of the *cis*-elements of *StALKBH* were revealed, among which the MYB element (57) was the most prevalent in the promoters, and the ABRE was predominantly present in the *StALKBH10C* promoter. Considering that promoters play various roles, the recognized *cis*-elements were classified into three categories, namely, hormone-responsive, plant development-responsive, and stress-responsive elements, accounting for 40%, 36%, and 24%, respectively, suggesting that the *StALKBHs* could participate in the response of potato to various phytohormones and stress stimulation.

### 2.5. Tissue Expression of StALKBH Family Genes

**Figure 5:**
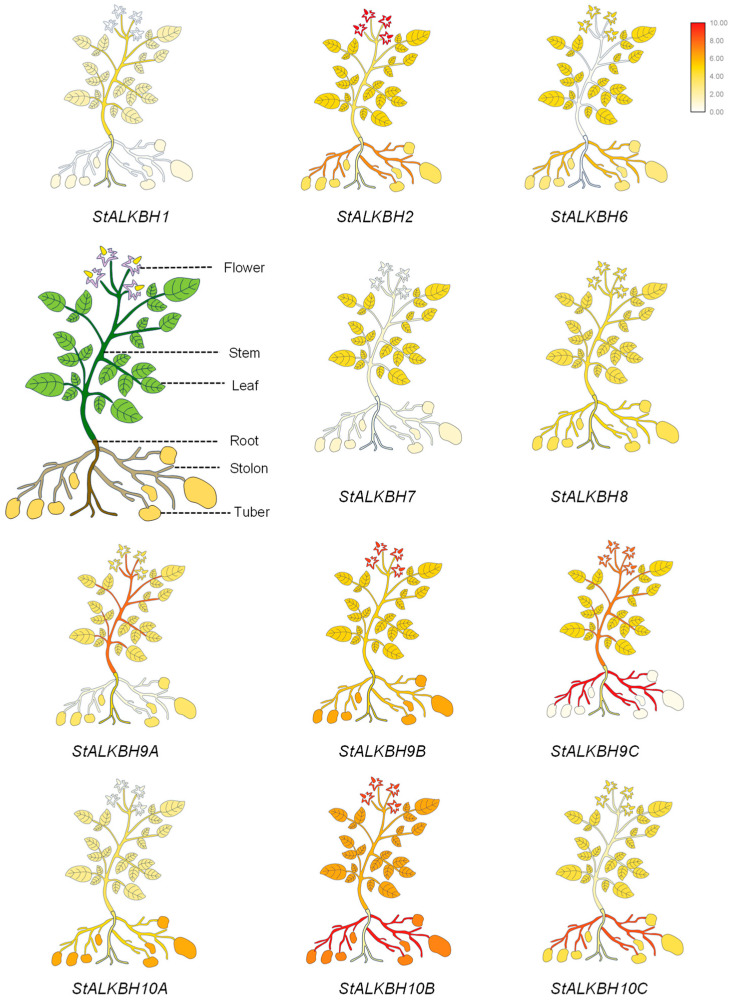
Analysis of *StALKBH* gene expression patterns in different tissues of potato. A heatmap of *StALKBH* expression data in six 30-day-old tissues (root, stem, stolon, tuber, flower, and leaf), created using TBtools.

To explore the expression patterns of the *StALKBH* family members in potato, qRT-PCR was conducted on tissues of the whole plant (Figure 5). A heatmap was constructed for the obtained expression data of six different potato tissues (root, leaf, bud, flower, stem, stolon, and tuber). Most of the examined genes exhibited the expression condition, and the expression profiles revealed that these genes potentially participated in the growth and development of potato. Compared with the other genes in the *ALKBH* family, *StALKBH1* and *StALKBH7* both displayed lower expression levels, suggesting that these two genes may not have a potential function or a specific temporal and spatial expression pattern. Furthermore, other *StALKBH*s exhibited tissue-specific expression and a significant change trend; among them, *StALKBH10B* and *StALKBH9C* showed a predominant expression trend, and they had higher levels of expression in the stolon than the other genes, indicating that they may be involved in stolon development and tuber construction. Additionally, *StALKBH9B* was observed to have a notably upregulated expression level in the flower, which suggests that *StALKBH9B* plays a role in potato reproductive development. Together, these results indicate that these genes may co-regulate the growth and development of potato.

### 2.6. Analysis of StALKBH Family Genes under Abiotic Stress Treatments

**Figure 6:**
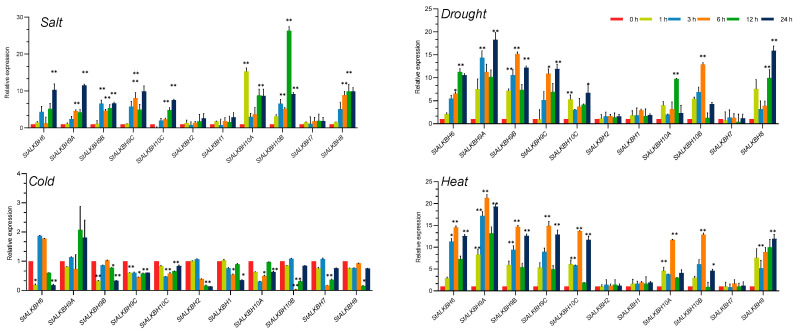
Analysis of *StALKBH* gene expression under different phytohormone treatments. Stress treatments: salt (150 mM), drought (20% PEG6000), heat (40 °C), and cold (4 °C). Each value represents the mean ± SE of three replicates, and the asterisks represent significant differences between the test group and the control group (* *p* < 0.05, ** *p* < 0.01; Student’s *t*-test).

Recently, growing evidence has demonstrated that m^6^A modification is involved in plant responses to various abiotic stresses, in which the mechanisms mediated by the *ALKBH* family members merit more attention. In the present study, we selected four abiotic stress treatments, namely, heat, cold, salt, and drought, to investigate the response of the *StALKBH* family members (Figure 6). In general, they could respond to multiple abiotic stresses, mostly exhibiting upregulated trends. For example, eight members (*StALKBH6*, *StALKBH8*, *StALKBH9A/B/C*, and *StALKBH10A/B/C*) were notably enhanced in all four stress treatments, with the max values of *StALKBH10B* and *StALKBH9B* expression being observed in the salt stress treatment. Additionally, gene expression was not suppressed after 12 h of salt treatment. These results indicate that *StALKBH*s may play positive roles in the tolerance to different abiotic stresses in potato. However, most of the genes displayed a notably downregulated expression in the cold treatment, except for *StALKBH9A*, in which only slight changes were induced. Thus, the above variations in the *StALKBH* family members with the different treatments suggest that this gene family is involved in complex abiotic stress responses in potato through the m^6^A methylation mechanism.

### 2.7. Analysis of StALKBH Family Genes under Hormone Treatments

**Figure 7:**
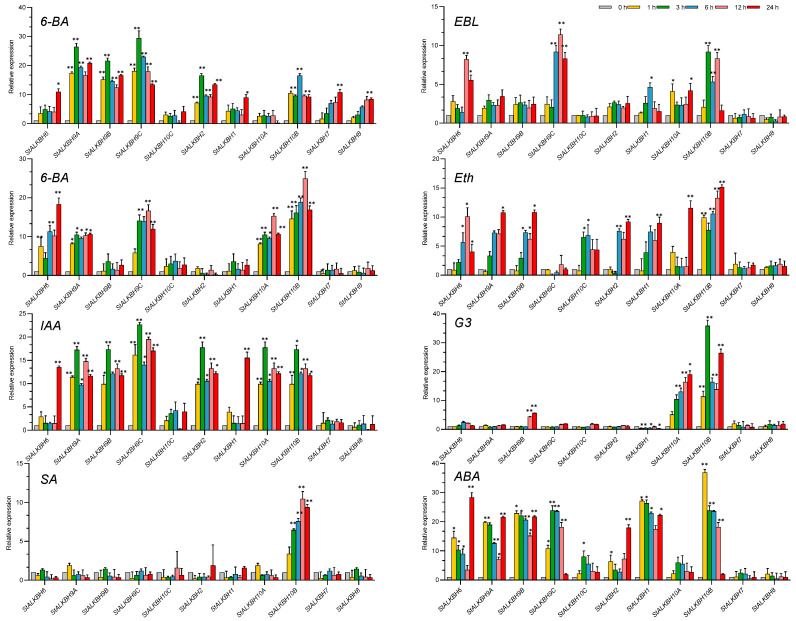
Analysis of *StALKBH* gene expression under different stress treatments. Each value represents the mean ± SE of three replicates. The potato leaves were collected after 0, 1, 3, 6, 12, and 24 h of treatment. Hormone treatments: 20 μM GA3, 20 μM IAA, 50 μM MeJA, 20 μM SA, 100 μM ABA, 10 μM 6-BA, 20 μM ethephon, 0.5 μM EBL. The asterisks represent significant differences between the test group and the control group (* *p* < 0.05, ** *p* < 0.01; Student’s *t*-test).

Phytohormones have been widely researched because they are essential for modulating plant growth and development. Therefore, the response to plant hormones must be examined when analyzing genome-wide family functions. First of all, various degrees of change were observed in *StALKBH* expression in treatments with eight major phytohormones: indole-3-acetic acid (IAA), 6-benzylaminopurine (6-BA), gibberellin A3 (GA3), ABA, ethephon, epi-brassinolide (EBL), MeJA, and salicylic acid (SA) (Figure 7). All of the *StALKBH* family genes responded to at least one plant hormone while exhibiting various response patterns to different plant hormones. For instance, the expression of *StALKBH10B* was significantly enhanced in response to the eight plant hormones, while that of *StALKBH7* and *StALKBH8* was only notably induced in response to 6-BA. Meanwhile, SA only influenced the expression of *StALKBH10B*, while no significant changes were observed in the other genes. The *StALKBH* genes could also be regulated, as opposite trends were observed in response to several phytohormones. For instance, GA3 induced the expression of most *StALKBH*s but repressed that of *StALKBH1*. However, ABA and MeJA positively affected all members of the *StALKBH* family, with the highest expression level of *StALKBH10B* being observed after 6 h of ABA treatment and an extreme value being observed after 12 h of MeJA treatment. This indicates that these genes may participate in the signaling of these two hormones, which may perform similar coordination and regulation functions to those of the *StALKBH* genes in plant development. To sum up, the diverse expression patterns of *StALKBH*s in several plant hormone treatments imply that these genes are involved in multiple hormonal signals in a complex manner. The specific interaction of these family members in phytohormone crosstalk may provide new insights into this field, which still requires further research.

## 3. Discussion

Initially, studies related to m^6^A methylation modification were more focused on writers; however, with the progress in research, it was gradually revealed that erasers have a regulatory function in gene expression at the post-transcriptional level, thereby playing key roles in various development processes [7,20]. The genome-wide identification of the *ALKBH* gene family is an essential step for comprehensively revealing potential mechanisms, and it has been performed in many species, including *Solanum lycopersicum* [21], *Populus* [22], and *Citrus sinensis* [24]. In the present study, 11 *StALKBH*s were identified in a BLASTP analysis of the potato genome with two other species (tomato and *Arabidopsis*) obtained from the phytochrome database, and a phylogenetic tree was constructed to analyze the evolutionary relationships among the plant kingdoms. Hmm-search and SMART analyses revealed that all the StALKBHs possessed a conserved 2OG-Fe(II)-Oxy domain. Additionally, the *StALKBH* members were also found to have similar gene structures (exon/intron) and conserved motifs. Protein sequences containing highly consistent amino acid sequences, particularly in functional domains, tend to share similar biological functions. The catalytic activity of ALKBH demethylase depends on Fe^2+^, and StALKBHs may mediate the oxidative demethylation of nucleic acids [22,25]. In biological evolution, genetic evolution is considered one of the important driving forces for gene duplication; moreover, the three predominant evolutionary patterns displayed in land plants are segmental duplication, tandem duplication, and transposition events [26,27]. *StALBH10B* and *StALKBH7* were adjacently located on the end of chr09, which may have been due to tandem duplication (Figure 1A). Besides tandem duplication, our synteny analysis also revealed the occurrence of segmental gene duplication. Two segmental duplication events occurred, *StALKBH9B–StALKBH9C* and *StALKBH10A–StALKBH10B*, indicating that these two pairs of genes may possess similar biological functions (Figure 1B). These results reveal the dynamic expansion of the *StALKBH* gene family and its potential functional diversity or redundancy in potato. Thus, a comprehensive identification and analysis of the *StALKBH* gene family helps to reveal the functions and mechanisms of m^6^A during plant growth and development.

Demethylase erasers have been discovered and identified to play a role in the m^6^A methylation mechanism; thus, this mechanism is regarded as a dynamic equilibrium mechanism [5,7]. ALKBH-mediated RNA demethylation involving eraser proteins has been considered an indispensable part of the epigenetic regulatory network for plant growth, development, and abiotic stress responses [2,4]. Furthermore, we investigated the expression of the *StALKBH* family genes in various potato tissues, among which *StALKBH9B* was found to have the highest expression level in the flower. In a similar study, *SlALKBH9B* (named *SlALKBH2* in this report) was considered to have the ability to bind *SlDML2* transcripts to modulate their stability in order to facilitate fruit ripening [14]. Remarkably, evolutionary and structural analyses revealed an apparent co-evolutionary relationship in the *StALKBH* family, in which the segmental duplication pair StALKBH9B–StALKBH9C was found, implying that these genes may possess an equivalent methylation function. *StALKBH9* and *StALKBH10* were more similar in terms of evolutionary relationships, and *StALKBH10B* expression was higher in the flower. The same results were demonstrated in a study conducted in 2017, with ALKBH10B-mediated mRNA demethylation being found to play a role in the stability of target transcripts in order to influence floral transition [13]. It was revealed that, in rice, OsALKBH9 plays a pivotal role in tapetal PCD and pollen exine accumulation by regulating m^6^A demethylation and participating in flower development [28]. It remains unknown how ALKBH-mediated m^6^A methylation affects potato growth and whether it regulates the development of other organs; thus, further research is required.

Studies on m^6^A methylation modification in potatoes have been unclear; however, a recent study found that the overexpression of the human m^6^A demethylase FTO gene in potato was able to induce a substantial increase in field yield and biomass of about 50% [29], suggesting that ALKBH-mediated m^6^A methylation has the potential to significantly improve the growth, development, and yield of potato during production and cultivation. To further reveal the possible functions of *StALKBH*s, we analyzed the *cis*-elements on the 2kb promoter sequences of 11 genes, and we found that these genes may respond to phytohormones, plant development-related signals, and abiotic stress.

Regarding phytohormone treatment, the most apparent and specific change in *StALKBH* expression occurred in ABA treatment, in which *StALKBH10B* expression was significantly enhanced and demonstrated the highest level. A previous study found that *AtALKBH8B* plays a positive role in salt stress tolerance in *Arabidopsis* by enhancing ABA signaling [30]. In contrast, a recent report found that *AtALKBH10B* is a negative modulator in the ABA response pathway during seed germination in *Arabidopsis* [16]. Thus, it can be speculated that StALKBH proteins have the same regulation pattern in potato. The *StALKBH* family mediates crosstalk with plant hormone signaling, indicating that further studies should not only focus on the relationship between transcriptional regulation and m^6^A methylation. In a previous study examining abiotic stress treatment, it was reported that salt stress increased the expression of *ALKBH9* in *Arabidopsis* roots, whereas it decreased the expression of *ALKBH10A* under high-temperature conditions [3]. These results are different from those in our study, in which both salt and heat stress were found to notably upregulate *StALKBH9* and *StALKBH10* expression. Huong et al. found that an *alkbh6* mutant had a higher level of m^6^A methylation than Col-0 *Arabidopsis*, and it exhibited a higher salt, drought, and high-temperature stress tolerance [17]. Another study showed that an *alkbh10b Arabidopsis* mutant had a reduced osmotic and salt stress tolerance during seed germination and exhibited a salt-hypersensitive phenotype [16]. In cotton, the demethylase *GhALKBH10* could improve drought tolerance in the seedling stage by reducing the level of m^6^A modification and contributing to the reduction in the mRNA stability of the ABA signaling pathway genes and Ca^2+^ signaling-related genes [18]. The same results were found in our study: most *StALBH* genes were induced in response to drought and salt stress. Furthermore, a relationship between the *ALKBH* family members and cold stress modulation was rarely reported, but it was speculated that *StALKBHs* may adversely regulate cold stress tolerance.

## 4. Materials and Methods

### 4.1. Plant Material, Growth Conditions, and Hormone and Stress Treatments

The potato cultivar “Qingshu9” was obtained from the Institute of Biotechnology, Qinghai Academy of Agricultural and Forestry Sciences, China. The cultivation conditions were 16 h (22 °C)/8 h (19 °C) light/dark, with a continuous light irradiance of 200 µmol·m^−2^·s^−1^, measured using an HR-350 Light Meter (Hi-point^®^ Firearms, Mansfield, OH, USA), and a relative humidity of 60%. Potato seedlings were sown in pots (24 cm diameter) filled with soil for growth. To analyze tissue-specific expression, samples were collected from potato seedings (30 days old). Root, stem, stolon, tuber, flower, and leaf samples were collected from at least 6 healthy individual plants. All samples were frozen immediately and mixed thoroughly after being ground. Each tissue group included three independent biological samples, and three technical repetitions were performed for each sample using qRT-PCR.

### 4.2. Identification of StALKBH Genes and Synteny Analysis

In 2020, the improved chromosome-level genome annotation of DM V6.1 was released and published [31]. The genome data of potato, *Arabidopsis*, and tomato (*Solanum lycopersicum* L.) were downloaded from the Phytozome database (https://phytozome-next.jgi.doe.gov/ (accessed on 19 May 2024)). To comprehensively identify the *StALKBH* family members in the potato genome, first, the potato genome was scanned with the protein sequences of *Arabidopsis* and tomato using the BLASTP-BLASTP (in TBtools 2.089) algorithm. To not miss potential *StALKBH* members, we obtained the HMMER (hidden Markov model) file 2OG-FeII_Oxy_2 (PF13532) from the Pfam database, and we searched for *StALKBH* family members in the potato genome using a query (*p <* 0.001). The protein length, molecular weight (MW), theoretical isoelectric point (pI), instability index, aliphatic index, and grand average of hydropathicity (GRAVY) were calculated using ExPASy (https://web.expasy.org/protparam/ (accessed on 19 May 2024)). Subcellular localization was predicted using an online analysis tool, BUSCA (https://busca.biocomp.unibo.it/ (accessed on 19 May 2024)).

### 4.3. Phylogenetic Analysis, Gene Structure, Protein Motifs, and Promoter Sequence Analysis

The ALKBH protein sequences of potato, tomato, and *Arabidopsis* were input into MEGA10 software to construct a phylogenetic tree using the neighbor-joining (NJ) method, with the bootstrap value set to 1000 replicates, and an improved visualization was obtained using iTOL (https://itol.embl.de/ (accessed on 19 May 2024)). The exon/intron structures of the *StALKBH* genes were recognized using the gff3 data of the potato genome. A conserved motif prediction analysis was conducted using MEME software (https://meme-suite.org/meme/index.html (accessed on 19 May 2024)) to identify the StALKBH protein sequences with the following parameters: any number of repetitions, a maximum of 10 misfits, and an optimum motif width of 6–200 amino acid residues. Multiple protein sequence alignment was displayed through DNAMAN. The domain patterns of the StALKBH proteins were visualized using the Pham batch search program (https://pfam.xfam.org/ (accessed on 19 May 2024)). The promoter response elements were detected using 2000 bp promoter sequences upstream of ATG on the PlantCARE server (https://bioinformatics.psb.ugent.be/webtools/plantcare/html/ (accessed on 19 May 2024)). All the sequence analyses of the above were visualized using TBtools software [32].

### 4.4. Chromosomal Location and Syntenic Analysis

The chromosome length and location of the *StALKBH* genes were displayed in the potato genome-related files using TBtools software. One-Step MCScanX was used to predict the synteny between the *ALKBH* genes in potato, *A. thaliana*, and tomato with the genome annotation and genome sequence files. The multiple synteny plot generated using MCScanX in TBtools software (version 2.089) was used to visualize synteny for the interspecific and intraspecific collinearity relationships of the *ALKBH* family members.

### 4.5. Hormone and Stress Treatments and Quantitative Real-Time PCR (qRT-PCR)

For the hormone treatments, the 30-day-old wild-type potato seedlings were treated with hormones such as 20 μM GA3, 20 μM IAA, 50 μM MeJA, 20 μM SA, 100 μM ABA, 10 μM 6-BA, 20 μM ethephon, or 0.5 μM EBL through external spraying for 0, 1, 3, 6, 12, or 24 h. The control seedlings received no treatment. After treatment, the seedlings were quickly frozen with liquid nitrogen and stored at −80 °C until use.

For the abiotic stress treatments, drought and salt stress treatments were carried out on the potato plants using solutions containing 20% (*m*/*v*) PEG6000 and 150 mM NaCl, respectively, followed by cultivation under standard conditions. The cold and heat stress treatments were conducted at 4 °C and 40 °C, respectively.

Total RNA was extracted with an RNAprep Pure Plant Kit (Vazyme Biotech, Nanjing, China), according to the manufacturer’s instructions. Complementary DNA (cDNA) was synthesized using a HiScript 1st-strand cDNA Synthesis Kit (Vazyme Bio Inc., Nanjing, China), according to the manufacturer’s instructions. All cDNA samples were diluted to the same concentration as the RT-qPCR analysis template. Specific ALKBH gene primers were designed, and sequence information is provided in Table S1. TB Green^®^ Premix Ex Taq™ II (Tli RNaseH Plus) (Takara, Osaka, Japan) was used to conduct qRT-PCR on a CFX96 Touch™ Real-Time PCR Detection System (BIO-RAD, Hercules, CA, USA). The relative expression levels were calculated using the 2^−∆∆Ct^ method. The expression levels of the different sampling cycles were normalized with the ef1α gene.

## 5. Conclusions

This study presents a comprehensive and systematic analysis of the *StALKBH* gene family members in potato. A total of 11 genes were identified and renamed to better understand their underlying functions according to the *Arabidopsis* genome. The chromosomal distribution and the synteny relationships, phylogenetic relationships, expression patterns, and *cis*-elements of the promoters of the *StALKBH* genes were analyzed. Comparative phylogenetic tree analyses among tomato and *Arabidopsis* were conducted, which classified the identified genes into seven groups. The expression patterns showed that most of the genes were extensively expressed in various tissues. Additionally, *StALKBH* gene expression responded to multiple phytohormone and abiotic stress treatments. Furthermore, our bioinformatics and evolutionary analyses will be helpful for better understanding the underlying evolutionary relationships and provide a foundation for further investigations into the functional properties of the *ALKBH* gene family in potato.

## Figures and Tables

**Table 1:** Physico-chemical characteristics of *StALKBH* family members in potato.

Gene Name	Gene ID	*Arabidopsis* Gene	CDS (bp)	Protein Length (aa)	Molecular Weight (KD)	pI	GRAVY	Subcellular Localization
*StALKBH6*	*Soltu.DM.01G016630.1*	*AT4G20350*	807	268	30.01	6.29	−0.17	Nucleus
*StALKBH9A*	*Soltu.DM.01G043090.1*	*AT1G48980*	1356	451	51.06	8.86	−0.57	Nucleus
*StALKBH9B*	*Soltu.DM.02G005900.1*	*AT2G17970*	1974	657	72.67	6.03	−0.57	Nucleus
*StALKBH9C*	*Soltu.DM.02G023950.1*	*AT4G36090*	1596	531	59.60	6.22	−0.65	Nucleus
*StALKBH10C*	*Soltu.DM.04G003750.1*	*AT1G14710*	1917	638	69.15	6.67	−0.49	Nucleus
*StALKBH2*	*Soltu.DM.04G010040.1*	*AT2G22260*	762	253	29.06	9.07	−0.64	Nucleus
*StALKBH1*	*Soltu.DM.04G016670.2*	*AT1G11780*	1065	354	39.88	5.74	−0.31	Nucleus
*StALKBH10A*	*Soltu.DM.06G034880.2*	*AT2G48080*	1545	514	56.77	8.06	−0.26	Nucleus
*StALKBH10B*	*Soltu.DM.09G022420.1*	*AT4G02940*	1647	548	60.64	5.97	−0.39	Nucleus
*StALKBH7*	*Soltu.DM.09G023290.2*	*AT4G02485*	780	259	29.39	4.69	−0.43	Nucleus
*StALKBH8*	*Soltu.DM.12G004160.1*	*AT1G31600*	1026	341	38.61	6.32	−0.40	Nucleus

## Data Availability

Data are contained within the article or Supplementary Materials.

## Supplementary Materials

The following supporting information can be downloaded at: https://www.mdpi.com/article/10.3390/ijms252010984/s1.
