# Supplementary material for: Genome-Wide Identification and Expression Analysis of the ALKB Homolog Gene Family in Potato (Solanum tuberosum L.)
Source: Int J Mol Sci. 2024 Oct 12;25(20):10984. doi: 10.3390/ijms252010984 (PMC11507222; doi:10.3390/ijms252010984)
Supplement: Supplementary file 1 [file ijms-25-10984-s001.zip › ijms-3198633-supplementary.pdf]

Supplementary_Material

Genome-Wide Identification and Expression Analysis of the ALKB Homolog Gene Family in Potato (*Solanum tuberosum* L.)

Yan Li [1,2,†] , Xuanming Dong [1,2,†], Jianyu Ma [3], Chenxin Sui [1,2], Hongju Jian [1,2,*] and Dianqiu Lv [1,2,*]

Table S1. The expression primer information of *StALKBH* family members in potato.

Primer	Sequence(5'-3')
StALKBH6-F	GCATCAAGACGGACCAGCT
StALKBH6-R	CACCAACAGGGCAGCTCA
StALKBH9A-F	GAAAGGGCCGTGTCACCA
StALKBH9A-R	CTAACAATGCCCGGCGGA
StALKBH9B-F	GGTGGTGCAGATCGGTCA
StALKBH9B-R	TTGTTCCTGGGCGCCAAT
StALKBH9C-F	TAGCGCCGCTGAGCAAAT
StALKBH9C-R	TCACACGTCCTTTGCCCC
StALKBH10C-F	GAGGTCCCGTCTCCCAGT
StALKBH10C-R	ACTGCGGACGAACACCTG
StALKBH2-F	TCGCGTCTTCGGTCGATC
StALKBH2-R	TGCATGAGGCTGGTATCCAC
StALKBH1-F	TGGAGCACCCTTGGCATG
StALKBH1-R	CAGGGTAGGTGCTGCCAT
StALKBH10B-F	GAGGTCGCCGACGTGAAA
StALKBH10B-R	CCACCACCTCACTGCCTG
StALKBH7-F	ACATGCAGCGCCCAAGAT
StALKBH7-R	GCGTGCTTCTCCCCACAT
StALKBH8-F	TTGGACCAGCTCACGGTT
StALKBH8-R	CATGATGCAAGGCCCTGC
StALKBH10A-F	GGCAACCTGGTGTCCCAA
StALKBH10A-R	AACCATCGGACGCACAGG

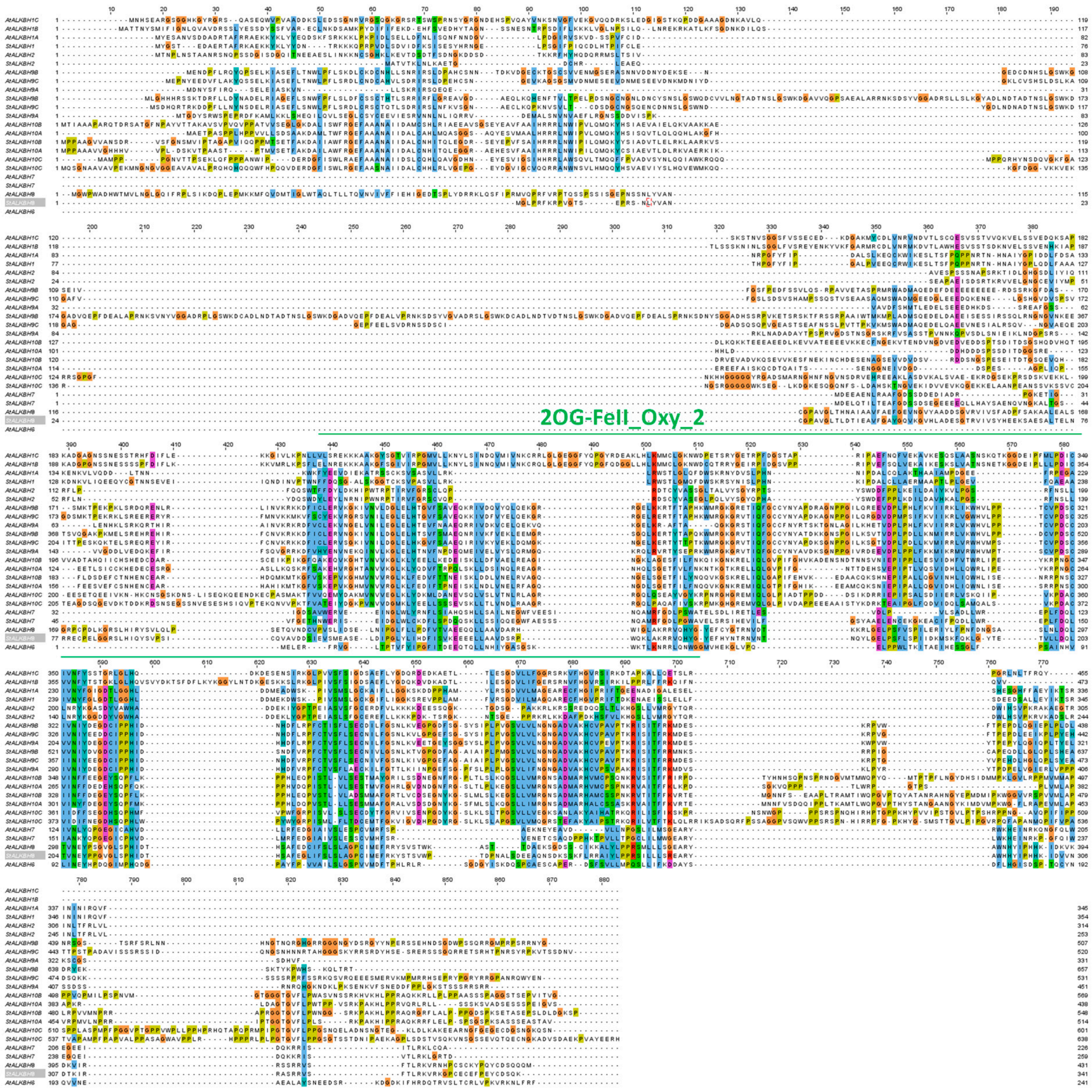

Figure S1. The *ALKBH* family protein sequence multiple alignment between *Arabidopsis* and potato.

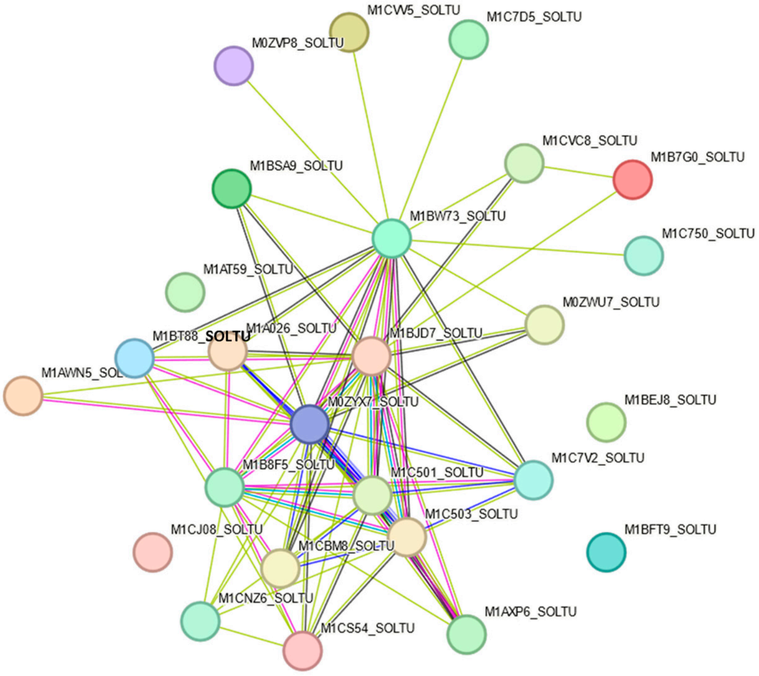

StECT1	M1C7V2_SOLTU
StALKBH6	M1CJ08_SOLTU
StFCA	M1BEJ8_SOLTU
StFIONA1	M1CS54_SOLTU
StECT7	M1CBM8_SOLTU
StALKBH9A	M1C750_SOLTU
StECT6	M1AXP6_SOLTU
StALKBH9B	M1C7D5_SOLTU
StCPSF30	M1BW73_SOLTU
StALKBH9C	M0ZVP8_SOLTU
StVIR	M1B8F5_SOLTU
StFIP37	M1BJD7_SOLTU
StALKBH10C	M1CVV5_SOLTU
StALKBH2	M1B7G0_SOLTU
StMTD	M1CNZ6_SOLTU
StECT5	M1C7V2_SOLTU
StMTC	M1C503_SOLTU
StMTB	M1C501_SOLTU
StALKBH10A	M1BSA9_SOLTU
StECT2	M1AXP6_SOLTU
StECT8	M1AXP6_SOLTU
StECT4	M1AXP6_SOLTU
StMTA	M0ZYX7_SOLTU
StHIZ2	M1AWN5_SOLTU
StHAKAI	M1BT88_SOLTU
StALKBH10B	M0ZWU7_SOLTU
StALKBH7	M1AT59_SOLTU
StCRY2	M1BFT9_SOLTU
StECT3	M1A026_SOLTU

Figure S2. Co-expression network of m^6A genes in potato. The edges/lines connecting the nodes represent the protein-to-protein interaction between genes.
